# Development and Evaluation of Intelligent Serious Games for Children With Learning Difficulties: Observational Study

**DOI:** 10.2196/13190

**Published:** 2020-04-16

**Authors:** Andrej Flogie, Boris Aberšek, Metka Kordigel Aberšek, Cecilia Sik Lanyi, Igor Pesek

**Affiliations:** 1 Anton Martin Slomsek Institute Maribor Slovenia; 2 Faculty of Natural Sciences and Mathematics University of Maribor Maribor Slovenia; 3 Faculty of Education University of Maribor Maribor Slovenia; 4 Faculty of Information Technology University of Pannonia Veszprem Hungary

**Keywords:** serious games, experimental, social skills, cognitive competence, intellectual disability, learning disabilities

## Abstract

**Background:**

Positive results can be obtained through game-based learning, but children with physical disabilities have fewer opportunities to participate in enjoyable physical activity. Because intelligent serious games can provide personalized learning opportunities, motivate the learner, teach 21st-century skills, and provide an environment for authentic and relevant assessment, they may be used to help children and adolescents with different kinds of learning disabilities to develop social and cognitive competences.

**Objective:**

The aim of the study was to produce and evaluate a suite of intelligent serious games based on accessible learning objectives for improving key skills, personal development, and work sustainability among children with learning difficulties.

**Methods:**

We conducted this research between 2016 and 2018, with pupils aged 11 to 12 years with learning disabilities who were integrated into the mainstream educational system. We used a 4-step methodology to develop learner creativity and social competences: (1) needs analysis, (2) development of learning content, (3) development of intelligent serious games, and (4) a usability evaluation focusing on the research questions and hypothesis. This was based on an initial teachers’ evaluation, using a survey, of students using 2 of the games, where the main goal was to determine user motivation and initiative and to improve the games and the evaluation process. The initial evaluation was followed by a pilot evaluation, which was performed for all proposed games, in all partner countries.

**Results:**

In an initial evaluation with 51 participants from Slovenia consisting of a pretest, followed by intelligent serious game intervention and concluding with a posttest, we observed statistically significant improvement in social and cognitive competences measured by tests. Based on these findings and observations, we improved the games and evaluation process. In the pilot test, conducted in all participating countries on a sample of 93 participants, the mean score on the teachers’ observation form on the pretest (before students began using the intelligent serious games) was 3.9. In the posttest, after students had used intelligent serious games, the mean score was 4.1.

**Conclusions:**

We focused on developing and evaluating intelligent serious games for persons with learning disabilities, particularly for students with disabilities who are integrated into the mainstream educational system. Such games provide an opportunity for personalized learning and should be tailored to ensure that every learner can achieve the highest standard possible. However, we recommend that the games be adapted based on the students’ needs and capabilities and a specially developed curriculum. The collected feedback showed that (1) children with learning disabilities need appropriately developed intelligent serious games, and (2) intelligent serious games, and the pertaining didactic methodology, should be based on an interoperable curriculum, so that teachers and trainers can use them. The student survey confirmed improvements in all aspects.

## Introduction

### Background

John Amos Comenius advised teachers to organize lessons into easily assimilated steps to make learning gradual, cumulative, and pleasant [1,2]. He emphasized the significance of play as a pedagogically effective activity. Today, Comenius’s ancient motto, *schola ludus,* has found new meaning in the modern use of interactive educational programs that use play and games as pedagogical tools [3]. The school-by-play philosophy was probably most importantly marked by the contributions of positive psychologist Martin Seligman [4] and the constructivist theorists Lev Vygotsky [5] and John Dewey [6,7]. Seligman described three kinds of happiness, which are also important in game-based learning: pleasure and gratification, embodiment of strengths and virtues, and meaning and purpose [4]. Playing games, including intelligent serious games (ISGs), has all the attributes needed for “flow,” according to Seligman and Csikszentmihalyi [8]. Playing computer games is a challenging activity that requires skill; it contains action and demands awareness; it has clear goals; and provides the player with immediate feedback. A well-designed game transports its players to their personal *flow zones*, delivering genuine feelings of pleasure and happiness [9].

Many studies have shown that positive results can be obtained through gamification and game-based learning. However, Malone et al [10] pointed out that people with physical disabilities have fewer opportunities to participate in enjoyable physical activity. One option for increasing physical activity is playing active video games. Their research examined energy expenditure and enjoyment in adults with mobility impairment during play and demonstrated positive results. Barnes and Prescott [11] and Dunn et al [12] also hypothesized that virtual reality platforms could be used for pediatric hemophilia care, allowing clinician orchestration, and being safe and feasible for use in distraction during procedures performed as part of complex health care. All these cases describe basic approaches to the notion of ISGs, and most reference a relatively narrow target group.

The term serious game means a game designed primarily for educational purposes rather than purely for entertainment [13-15]. Intelligent game–based learning environments integrate commercial game technologies with artificial intelligence methods derived from intelligent tutoring systems and intelligent narrative technologies. ISGs can provide personalized learning opportunities, offer more motivation and engagement for the learner, teach 21st-century skills, and provide an environment for authentic and relevant assessment [16-18]. It is important for the player or learner in this context that negative consequences are not typically associated with failure. Even more, failure is seen as a typical and integral part of playing, and of learning [17,18]. In the context of school curricula and subjects, gaming provides an excellent opportunity for formative assessment [19,20]. Serious games are often mentioned as an important means for teaching 21st-century skills because they can accommodate a wide variety of learning styles and personalized learning within a complex decision-making context [21].

Personalized learning can be particularly important for students with disabilities who are integrated into the mainstream educational system. However, serious games should be adapted based on student needs. In traditional classroom settings, a student who has not grasped a concept could end up with a gap in their knowledge base, whereas serious games inherently force the player to grasp a concept in order to advance. Players can repeat the same scenario until they have grasped the concept [13,22]. This justifies the placement of serious games in the context of constructivist theory: they are considered similar to Vygotsky’s zone of proximal development, which is “the distance between the actual developmental level as determined by independent problem solving and the level of potential development through problem solving under adult guidance or in collaboration with more capable peers” [5,13].

Several research projects focusing on the learning process in people with impairments (mental or sensory) have revealed that ISGs are an excellent didactic tool for reaching educational goals. The work of Carvelho et al [23] confirmed positive results in a population with visual disabilities, and Baker [24] achieved similar encouraging results working with people with autism. Schneider et al [25] also worked with persons with dyslexia. Brown et al [26] proved the effectiveness of combining ISGs with mobile apps, which can be used anywhere, to reach a higher level of independence for persons with Down syndrome. Barnes and Prescott [11] proved that therapeutic games create clinically measurable reductions in symptoms of anxiety in adolescents. Dunn et al [12] reported that serious games can provide a distraction during medical procedures.

Serious games have several advantages when used as a tool in the educational process for children with learning disabilities. According to Connolly et al [27,28], children with disabilities who are commonly integrated into mainstream school environments often feel uneasy. For them, ISGs are an interactive way of modelling and reinforcing positive behaviors. Such games help players learn how to interact with the world in safer and more controlled environments, where challenges can be gradually introduced [29,30]. A possible disadvantage of using ISGs is that they can result in a lack of interest in studying. Moreover, they can have hidden risks for students: while computers are an invaluable educational tool, they can also be a source of problems and can diminish the overall value of in-person education. Ke and Abras [31] suggested that game challenges should be open ended and allow for partial success. Game designers should also embed scaffolding features to assist recall, reflection, and metacognitive regulation to support students with special learning needs.

However, proper implementation might help keep the drawbacks to a minimum. Better planning is necessary [29,32-34]. Based on the theory of ISGs, and on feedback from the survey conducted as part of this project, we began the design of ISGs.

### Objectives

This research focused on serious games, including the process of gathering requirements, as well as the design and implementation of such games, as applied in the project Intelligent Serious Games for Social and Cognitive Competence (ISG4competence) [35]. It involved the participation of 3 universities and 4 companies, from Turkey, Slovenia, Hungary, Bulgaria, and Belgium. The main goal of the project was to develop a didactic concept and, on that basis, to produce ISGs for improving social and cognitive competences of children with learning difficulties. More specifically, the aim was to help children with learning disabilities in developing creativity, social skills, cognitive skills, and work skills. Using ISGs and 3D simulations helps these children in their process of social integration and personal development [36]. In the project, we used ISGs and 3D simulations to make teaching and learning more interesting, playful, attractive, and efficient [37].

## Methods

### Study Design

We approached our main goal, to develop learner creativity and social competences, through a 4-step process. The research was conducted between 2016 and 2018, in lower secondary schools with pupils aged 11 to 12 years. We focused on students with learning disabilities who were integrated into the mainstream educational system.

The aim of the study was to produce and evaluate a suite of ISGs based on accessible learning objectives for improving cognitive skills, personal development, and work sustainability among children with learning difficulties. Table 1 presents the instruments used to measure outcomes and the methodological process for the study [35].

**Table 1:** Methodological process of the study.

Step	Methods	Instruments	Products
1. Needs analysis	Survey	Questionnaire: Q1-Q16 (Multimedia Appendix 1)	Survey report
2. Development of learning content	Development of curricula and scenario framework	N/A^a^	Curricula and scenario framework [35]
3. Development of intelligent serious games	Development of games	N/A	10 games, 2 examples
4. Usability evaluation	*Initial evaluation* in Slovenia Pre-evaluation Postevaluation *Pilot evaluation* Prepiloting Postpiloting	Observation form for teachers *Initial evaluation*: Q1-Q10 Questionnaire for students *Pilot evaluation*: Q1-Q16 (Multimedia Appendix 1)	Teacher trainer qualitative and quantitative report Evaluation report

^a^Not applicable.

In the first step, we designed a user needs survey to identify the context, for example, to analyze the target audience characteristics and learning and training needs. The second step was to develop the learning content and objectives based on user needs. The third step was to develop the games. The fourth and final step, which involved only children with disabilities who were integrated into mainstream schools, was to evaluate these games and their implementation. This pilot testing in the fourth step focused on testing the games’ usability; in future, we hope to receive user feedback, in order to eventually optimize and improve the games.

### First Step: Needs Analysis

We performed a needs analysis by means of per-country reports and a consolidated overall survey report on stakeholders and target groups in Turkey, Slovenia, Hungary, Bulgaria, and Belgium, resulting in a qualitative and quantitative analysis of findings (national and comparative). For the survey, we deemed both an online survey in the national language and face-to-face meetings and interviews to be appropriate tools for collecting the data we required. In the analysis of self-awareness and social awareness (as part of social competence), we also took into account the guidelines of the Trait Emotional Intelligence Questionnaire [38], that is, the guidelines for its short form (TEIQue-SF) [39,40]. According to the guidelines, we adapted the TEIQue-SF questionnaire to the needs of our study according to the table in Multimedia Appendix 1.

### Second Step: Development of Learning Content

Based on the needs analysis, we identified and created the required content where we defined the learning content, methods, and structure of the games, which was the basis of the next step.

### Third Step: Development of Intelligent Serious Games

The ISG4competence consortium agreed to develop ISGs [41] for desktop and mobile use based on user needs and learning content.

### Fourth Step: Usability Evaluation

#### Sample

The purpose of the initial evaluation was to test the proposed games in a real-life learning environment, focusing on the students’ motivation and concentration. Results served as the grounds for possible improvements to the games. The initial evaluation was followed by a pilot evaluation, which was performed for all proposed games, in all partner countries.

The pilot testing also involved trainers (educators) whose task was to train and guide students (ie, help them with game playing and with answering the questionnaires), and especially to evaluate and interpret the results.

#### Evaluation of the Intervention

The aim of the fourth step of the project was to provide a usability evaluation focusing on how the primary goals of ISGs were assessed (with regard to social competences and creativity), and whether the evaluation methods were suitable for assessing these goals. We evaluated and optimized the process in 2 steps. The aim of the *initial evaluation* was to check only some of the proposed ISGs in a real-life learning environment. This step focused especially on the appropriateness of the concept in relation to the students’ motivation for and concentration in working with ISGs. For this evaluation, we also prepared an observation form for teachers. We conducted prepilot testing only in Slovenia. The *pilot evaluation* was designed to test the games’ usability on a wider sample, that is, all partner countries. The purpose was to receive feedback from the users for eventual improvement and optimization of the games, with a view to incorporating end users in the process of game development.

For both evaluations, the materials included surveys. All questions in the initial and pilot evaluations were included in the pre-evaluation and postevaluation surveys, with closed-ended questions, with answers selected from a 5-point Likert scale. Multimedia Appendix 1 presents the questions. The survey consisted of recording sheets for students, which gathered information on the impact of ISGs, and an impact assessment survey for measuring the status of creativity and cognitive competences in students with learning difficulties in mainstream schools. These were aimed at ensuring that ISGs were repeatedly tested throughout the development cycle, to make sure they met their core aims.

The evaluation was conducted during classes and extracurricular activities, that is, during after-school programs (after regular classes, students can stay in school to perform various activities, including homework, remedial education, or self-tuition). The evaluation was performed for a period of 1 month. After classes and with the support of teachers (who were trained during the first week), the games were used in various learning situations for 3 consecutive weeks, approximately twice a week. The students then continued playing (testing) the games during after-school programs, partly still supported by the teachers, on average 3 times per week. They were also able to download the games onto their computers and play them at home, both individually (offline), and some of them (eg, Minecraft: Education Edition) with their classmates (online). In these cases, the choice of game and the number of repetitions were not monitored.

## Results

### First Step: Needs Analysis

The sample for the needs analysis comprised the following groups: 100 participants from Turkey, 78 participants from Slovenia, 92 participants from Hungary, 110 participants from Bulgaria, and 105 participants from Belgium. The respondents came from a wide range of target groups, including people with learning disabilities, parents and teachers of children with disabilities, nongovernmental organizations, special school educators, special education trainers, and training providers [35].

#### Significant Findings

The following 5 findings from the survey were relevant to the development of ISGs in the ISG4competence project [35].

First, formal definitions of children with learning problems and difficulties differed among countries, but in practice the same target groups were identified, while the degree of inclusive education varied considerably among the participating countries.

Second, pedagogical methodologies to support the acquisition of social competences and creativity were, in general, highly diversified, and often depended on the needs of specific target groups.

Third, there was a clear willingness to introduce ISGs into teaching environments; however, restrictions did exist, mainly owing to a lack of financing for equipment, bureaucratic issues with the process of providing permission for their implementation in mainstream schools, and a lack of time.

Fourth, learning challenges faced by children with learning difficulties were similar in all participating countries, with regard to the educational and social levels. The use of ISGs was generally limited. There was, for example, a negative correlation between the use of games and the size of the school: the bigger the school, the fewer ISGs were used.

Fifth, a wide range of pedagogical approaches (including game playing) were applied to the different beneficiary groups in all participating countries. The individual approach, which is generally recommended, is a challenge, given a lack of time and financial resources.

The effectiveness and efficiency of information and communication technology educational tools require considerable effort by the trainer or educator. Solutions should, therefore, consider providing tools for training the trainers.

Based on these findings, we produced the Curriculum and Scenarios Framework document, which can be downloaded (Intellectual Output 2) from the project website [35]. This served as a basis for the second step, that is, developing learning content.

#### Students’ Learning Disabilities

**Figure 1:**
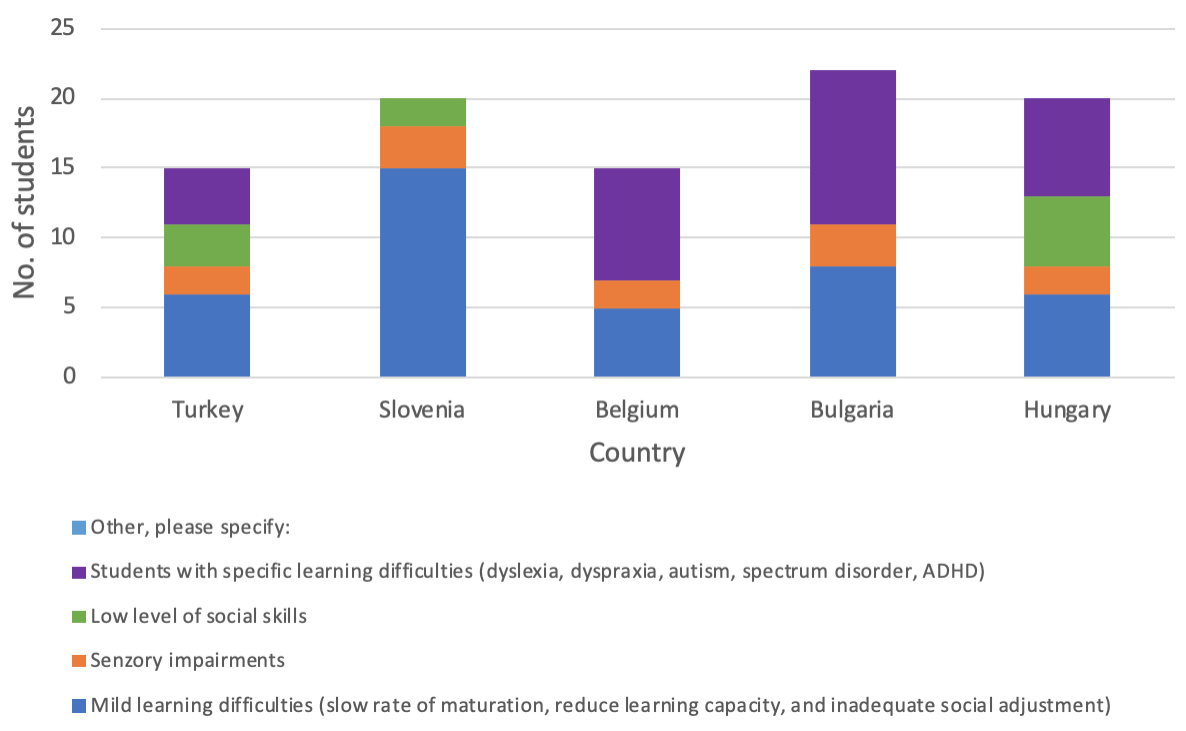
Distribution of student’s learning disabilities, by country. ADHD: attention-deficit/hyperactivity disorder.

Figure 1 shows the range of students’ learning disabilities, mainly identified as mild or specific learning, by country.

#### Why Existing Pedagogical Approaches and Training Materials Failed

Table 2 lists reasons why existing pedagogical approaches and training materials in partner countries failed to ensure successful acquisition of cognitive competences.

**Table 2:** Reasons for the failure of existing pedagogical approaches in different countries [35].

Reasons	Country				
	Turkey	Belgium	Bulgaria	Slovenia	Hungary
Lack of qualified staff, suitable application, adequate and effective process, adequate time, (financial) resources	X	X	X	X	X
Materials are not learner centered, technological approaches not sufficiently attractive or usable, class size too large	X	X	X	X	X
Inadequate prerequisite knowledge and skills, technological tool deficiencies	X	X	X	X	N/A^a^
Lack of assessment	X	X	N/A	X	N/A
App failure, environmental problems	X	X		X	X
Individual subjectivity or differences, individuality is secondary, one size does NOT fit all, lack of awareness of incomprehension	X	X	X	N/A	X
Family problems, not understanding behavioral deficiencies	X	N/A	N/A	N/A	X
Outdated methods	X	N/A	X	X	X
Children not given enough space to socialize	X	X	N/A	X	N/A
Inadequate research	X	N/A	N/A	N/A	N/A
Children were not assessed on an individual basis	X	N/A	X	N/A	X
Missing feedback	X	N/A	N/A	N/A	N/A
Teacher dependent	X	X	N/A	N/A	X
Acquisition of cognitive competences is a long and difficult process	N/A	X	X	N/A	X

^a^Not applicable.

#### Barriers to the Acquisition and Enhancement of Cognitive and Social Competences

**Figure 2:**
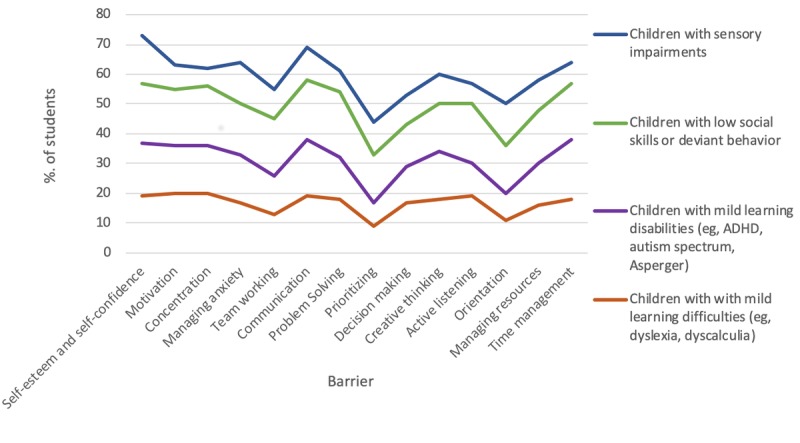
Main barriers to acquiring cognitive competencies, by learning disability. ADHD: attention-deficit/hyperactivity disorder.

The main difficulties that hampered the acquisition of cognitive competencies were similar across participating countries, but the degree to which they affected the beneficiaries differed. The feedback revealed that children with sensory impairments faced the most difficulties, whereas children with mild learning disabilities faced the most varied kinds of difficulties, especially with regard to self-esteem and self-confidence, problem solving, and time management. Figure 2 shows some of the results.

#### Cognitive and Social Competence Tendencies

Students from all countries displayed similar tendencies in the cognitive and social competences that were to be achieved. In this question, students could select competences by choosing as many as they wished. When all data were consolidated, we counted the answers and to obtain the outcomes. Table 3 shows that the following social competences scored the highest: self-esteem and self-confidence, followed by communication, problem solving, concentration, teamwork, motivation, and active listening.

**Table 3:** Cognitive and social competences.

Competence	Number of students
Self-esteem and self-confidence	250
Motivation	211
Concentration	222
Managing anxiety	170
Team work	210
Communication	249
Problem Solving	233
Prioritizing	142
Decision making	150
Creative thinking	175
Active listening	205
Orientation	141
Managing resources	119
Time management	168
Other (please specify)	19

### Second Step: Development of Learning Content

In this step we prepared first the syllabus for all learning content and then the teacher manuals for the ISGs.

### Third Step: Development of Intelligent Serious Games

**Figure 3:**
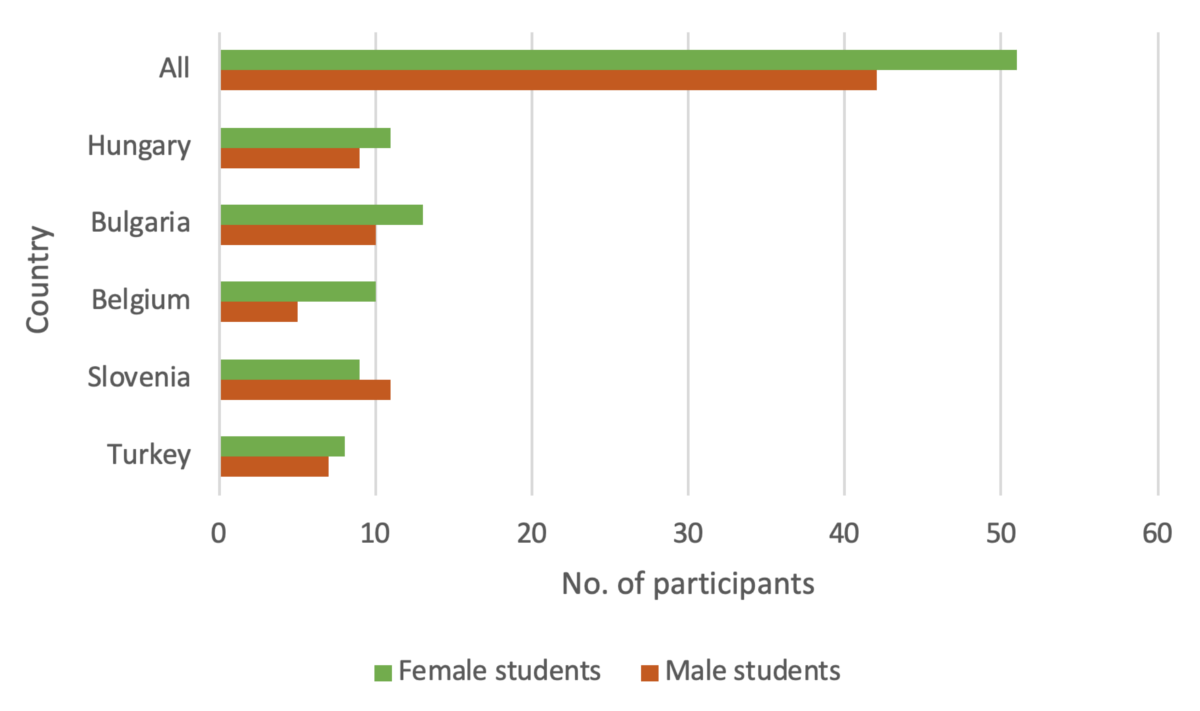
Distribution of the study sample for pilot testing by country and sex.

All games are available in English, Bulgarian, Dutch, Hungarian, Slovenian, and Turkish. We developed 10 games with the following titles: Math, Pair Cards, Labyrinth, Car Race, Manage Yourself, Sequence, Memory (designed to help with problems associated with a visual sequential memory deficit), Into the Forest (designed for children with speech disorders), VR Shop (a flash player game that runs on any Web browser), and Weekend Wonderland (the background theme of this story-based game [35] is a leisure park; the game offers many interesting and challenging tasks). All these games were played during the pilot phase in all partner countries. Figure 1 and Figure 3 show the characteristics of the players. Below, we discuss 2 examples in more detail.

#### Car Race

**Figure 4:**
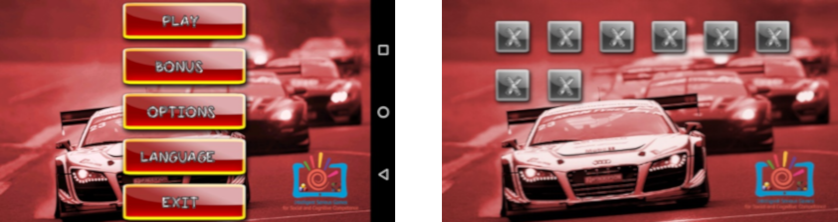
Screenshots of the Car Race game.

In this game, the user navigates the track by using 2 fingers to touch the screen (see Figure 4). One of the main advantages is that the user can navigate the game using only 1 hand, which means that it is suitable for children who can use only 1 of their hands. The game has 16 levels, 8 bonus levels, and an operating menu system; it is a side-view car app with realistic physical attributes.

Car Race was developed for the Android (Google LLC) platform using the AndEngine game engine (Nicolas Gramlich) and its plug-ins in the Eclipse Android Development Tools environment. The game is appropriate for classroom and individual use and can be downloaded for free [35]. Our research shows [35] that it is mostly useful as a tool for developing sensory motor functions, for managing anger and stress, and as a means of enhancing self-esteem, concentration, and motivation. It is suitable for users with mental and physical disabilities of any age group [42].

#### Minecraft: Education Edition

This is a version of the popular open world game Minecraft and is specifically designed for education to be a versatile, open platform; it can be used to teach all subjects, from mathematics and physics to history and languages [43]. By using the digital platform and classroom experience, students can develop social skills, collaboration, problem solving, communication, digital citizenship, and more. There is no limit to what students can learn in the game, and no limit to how the game can extend classroom learning.

Minecraft: Education Edition is specifically designed to enable teachers, trainers, and students to be creative in ways not possible in the real world. It has a social component, where students can cooperate and communicate in order to survive in the harsh conditions of Minecraft World. Working together helps students to build a positive classroom climate, to teach the benefits of collaboration, and to facilitate teamwork [31-43].

In the Minecraft: Education Edition, students enter the ISG4competence world, where they can develop various cognitive and social competences, such as problem solving, teamwork, and collaborative learning. We developed 3 main scenarios.

The first scenario addressed following instructions in order to solve a problem. The students are divided into 2 groups. The first group are builders and give instructions to the second group. The players in the second group try to build objects according to instructions from the first group. With this scenario, students are trained in two kinds of communication skills: to give clear instructions in the correct sequence (the first group), and to receive and follow the instructions (the second group). This communication competence is trained at 2 levels. At the first level, the students can ask additional questions if they did not understand the instruction or a part of the instruction. At the second level, they are required to understand the given instruction immediately and use this understanding, to solve the task.

In the second scenario, teamwork and team building, students are taught how to solve problems, relying on communication with and without feedback. They learn to follow instructions and ask questions, which helps to develop their cognitive and social competences.

In the fourth scenario, learning the basics of programming and robotics, students control their own virtual robot and guide it across various areas.

**Figure 5:**
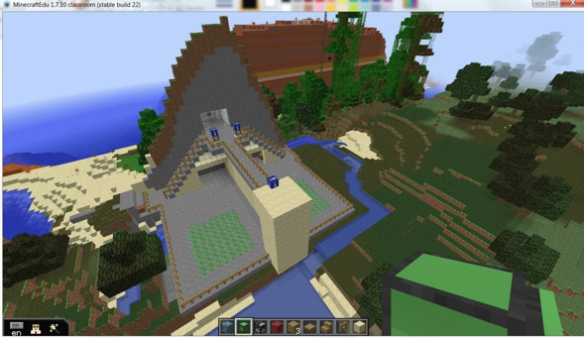
Screenshot of the ISG4competences world in Minecraft: Education Edition, for scenario 1 (following instructions in order to solve a problem).

Figure 5 presents the main playground for developing communication competences and problem-solving skills. Two groups are separated by a wall and must communicate to solve a problem.

Other serious games developed by the project team can be accessed and downloaded from the project website [35].

### Fourth Step: Usability Evaluation

We conducted the *initial evaluation* only in Slovenia and only on learners with mild learning disabilities in mainstream lower secondary schools, in the sixth and seventh grades, that is, students aged between 11 and 12 years. The sample of 51 participants consisted of 22 (43%) boys and 29 (57%) girls. All of them provided official documentation of their mild learning disability. Our initial evaluation thus involved a relatively homogeneous, randomly selected group.

In the *pilot evaluation*, 93 students participated. The study sample was fairly homogeneous (42, 45% male students and 51, 55% female students) as Figure 3 shows.

**Figure 6:**
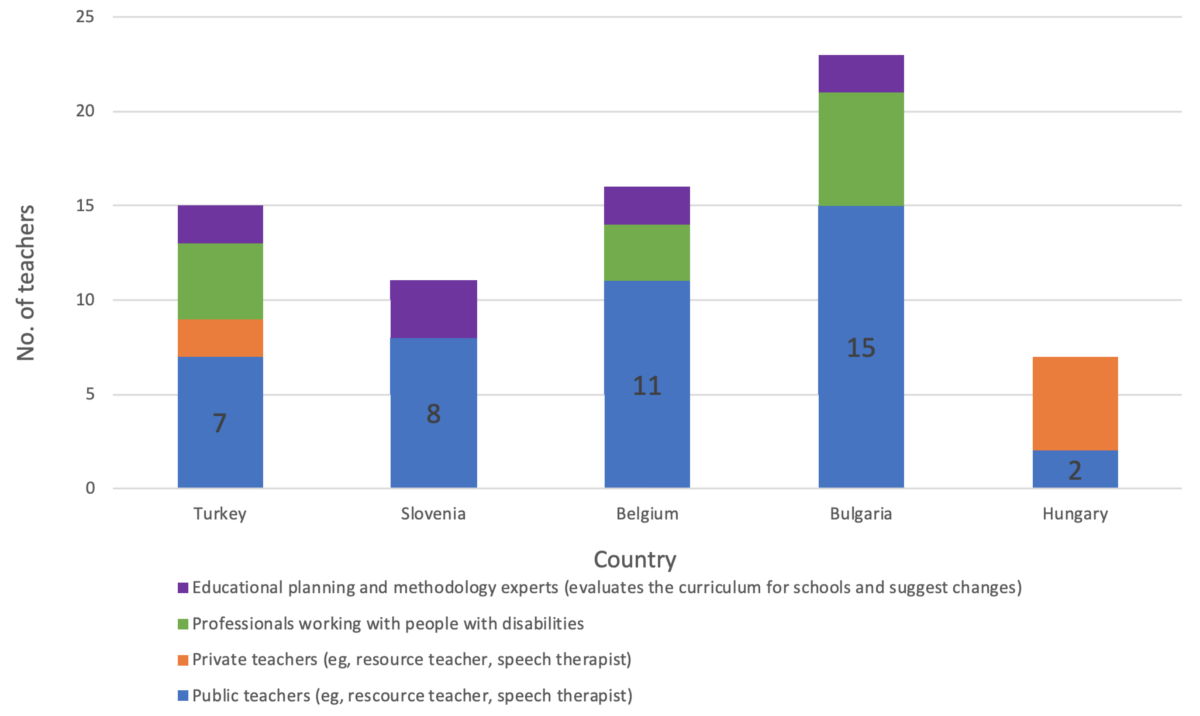
Distribution of the study sample teachers and trainers.

The pilot evaluation also involved 71 trainers (educators), the majority of whom were public school teachers. Their task was to train and guide students (ie, help them with game playing and with answering the questionnaires), and especially to evaluate and interpret the results. Figure 6 shows the structure of the teacher and trainer sample.

#### Initial Evaluation

We conducted the preliminary evaluation of the games we had developed only in Slovenia. This served as the basis for improvement and optimization of the proposed ISGs, with an emphasis on students’ motivation and concentration, using 2 of the games that are presented in this paper. In the initial evaluation, we used an observation form for teachers, in which they reported on the students (n=51) before and after they began using the ISGs for developing creativity and social competences. Table 4 presents the results of the pre-evaluation.

Table 5 present the results obtained after students played several ISGs during the initial evaluation with the same 51 students.

**Table 4:** Results of the initial pre-evaluation (N=51).

Questionnaire items	Answer score^a^, n (%)						Score, mean (SD)	
	1	2	3	4	5	Total		
Q1. Interacts nonverbally with other children with smiles, waves, nods, etc.	36 (75)	4 (8)	5 (10)	3 (6)	1 (2)	49 (100)		1.6 (0.5)
Q2. Expects a positive response when approaching others.	36 (76)	4 (9)	4 (9)	2 (4)	1 (2)	47 (100)		1.5 (0.5)
Q3. Expresses wishes and preferences clearly; gives reasons for actions and positions.	30 (65)	7 (15)	4 (9)	3 (7)	2 (4)	46 (100)		1.7 (0.4)
Q4. Asserts own rights and needs appropriately.	33 (70)	5 (10)	5 (10)	3 (6)	2 (4)	48 (100)		1.6 (0.6)
Q5. Is not easily intimidated by bullying.	36 (76)	4 (9)	4 (9)	2 (4)	1 (2)	47 (100)		1.5 (0.5)
Q6. Expresses frustrations and anger effectively, without escalating disagreements or harming others.	38 (81)	2 (4)	4 (9)	2 (4)	1 (2)	47 (100)		1.5 (0.5)
Q7. Gains access to ongoing groups at play and work.	36 (75)	4 (8)	5 (10)	3 (6)	1 (2)	49 (100)		1.6 (0.4)
Q8. Enters ongoing discussion on a topic; makes relevant contributions to ongoing activities.	35 (74)	3 (6)	5 (10)	3 (6)	1 (2)	47 (100)		1.6 (0.5)
Q9. Takes turns easily.	32 (68)	5 (10)	4 (9)	4 (9)	2 (4)	47 (100)		1.7 (0.5)
Q10. Has positive relationships with one or two peers; shows the capacity to really care about them and miss them if they are absent.	29 (4)	7 (6)	7 (11)	3 (40)	1 (38)	47 (100		1.7 (0.6)

^a^Likert scale answer options were 1, “not at all;” 2, “a little;” 3, “somewhat;” 4, “mostly;” and 5, “a lot.”

**Table 5:** Results of the initial postevaluation (N=51).

Questionnaire items	Answer score^a^, n (%)							Score, mean (SD)
	1	2	3	4	5	Total		
Q1. Interacts nonverbally with other children with smiles, waves, nods, etc.	4 (8)	4 (8)	8 (17)	15 (31)	17 (35)	48 (100)	3.8 (1.3)	
Q2. Expects a positive response when approaching others.	1 (2)	4 (9)	14 (30)	20 (43)	8 (17)	47 (10%)	3.6 (0.9)	
Q3. Expresses wishes and preferences clearly; gives reasons for actions and positions.	0 (0)	3 (7)	9 (20)	24 (52)	10 (22)	46 (100)	3.9 (0.8)	
Q4. Asserts own rights and needs appropriately.	1 (2)	3 (6)	13 (28)	18 (38)	12 (26)	47 (100)	3.8 (1.0)	
Q5. Is not easily intimidated by bullying.	1 (2)	1 (2)	12 (26)	21 (45)	12 (26)	47 (100)	3.9 (0.9)	
Q6. Expresses frustrations and anger effectively, without escalating disagreements or harming others.	0 (0)	9 (19)	12 (26)	19 (40)	7 (15)	47 (100)	3.5 (1.0)	
Q7. Gains access to ongoing groups at play and work.	1 (2)	1 (2)	11 (23)	21 (45)	13 (28)	47 (100)	3.9 (0.9)	
Q8. Enters ongoing discussion on a topic; makes relevant contributions to ongoing activities.	0 (0)	5 (11)	6 (13)	25 (53)	11 (23)	47 (100)	3.9 (0.9)	
Q9. Takes turns easily.	1 (2)	3 (6)	4 (9)	19 (40)	20 (43)	47 (100)	4.1 (1.0)	
Q10. Has positive relationships with one or two peers; shows the capacity to really care about them and miss them if they are absent.	2 (4)	3 (6)	5 (11)	19 (40)	18 (38)	47 (100)	4.0 (1.1)	

^a^Likert scale answer options were 1, “not at all;” 2, “a little;” 3, “somewhat;” 4, “mostly;” and 5, “a lot.”

The initial evaluation revealed that the difference between the preplaying and postplaying levels of social competence was too great. Therefore, with a view to optimization, we prepared a special introductory program for all those who participated in the pilot evaluation. This introductory program provided guidelines for participation in the training, an explanation of the games, and a demonstration of how to play the games. Only after providing this program did we carry out the initial pilot evaluation.

#### Pilot Testing

**Figure 7:**
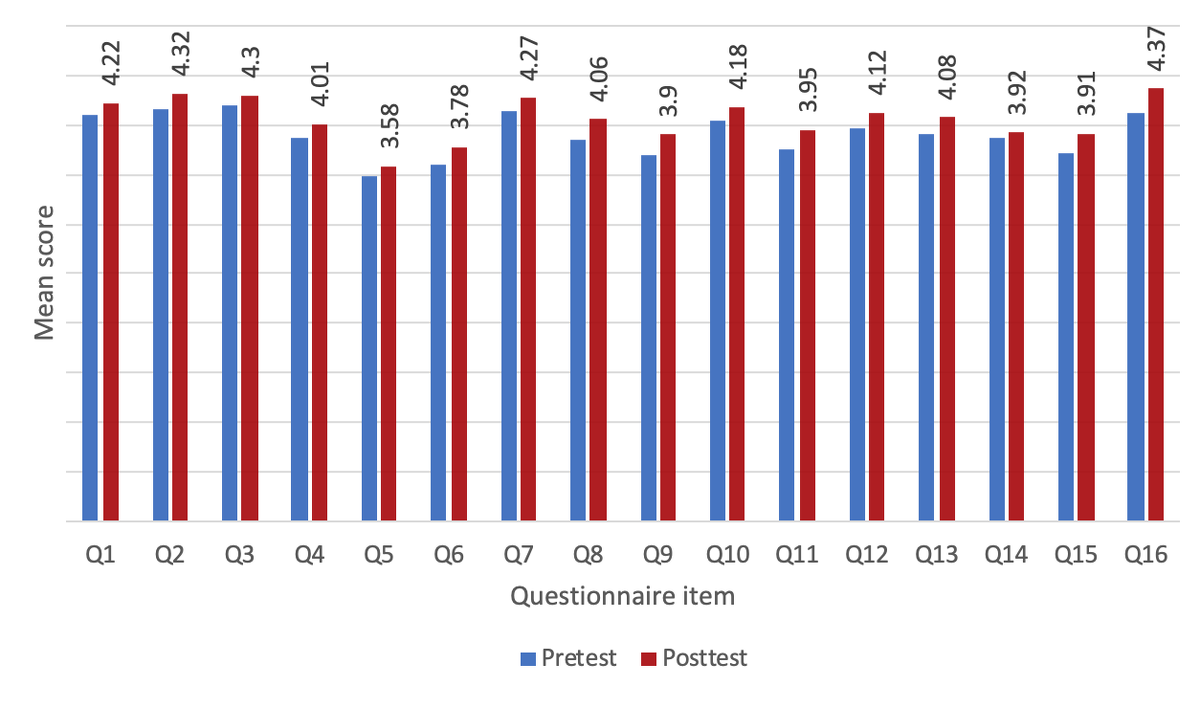
Results of questionnaire responses before (pretest) and after (posttest) final intelligent serious games testing.

In the pilot testing, 93 students were tested, coming from all participating countries. The survey was more detailed and contained 16 questions (Multimedia Appendix 1). Figure 7 shows the results.

Next, we applied a paired-sample *t* test, comparing the students’ answers from before versus after playing ISGs. Table 6 shows the results.

**Table 6:** Paired-sample *t* test (N=93).

Pair^a^	Correlation	*P* value	Cohen *d*
Pair 1 (Q1b-Q1a)	.906	.001	.35
Pair 2 (Q2b-Q2a)	.721	.002	.32
Pair 3 (Q3b-Q3a)	.435	.09	.18
Pair 4 (Q4b-Q4a)	.426	.02	.24
Pair 5 (Q5b-Q5a)	.689	.03	.23
Pair 6 (Q6b-Q6a)	.593	<.001	.46
Pair 7 (Q7b-Q7a)	.851	.001	.35
Pair 8 (Q8b-Q8a)	.58	<.001	.49
Pair 9 (Q9b-Q9a)	.522	<.001	.39
Pair 10 (Q10b-Q10a)	.776	<.001	.38
Pair 11 (Q11b-Q11a)	.698	<.001	.39
Pair 12 (Q12b-Q12a)	.871	<.001	.42
Pair 13 (Q13b-Q13a)	.857	<.001	.44
Pair 14 (Q14b-Q14a)	.583	.32	.10
Pair 15 (Q15b-Q15a)	.439	<.001	.39
Pair 16 (Q16b-Q16a)	.690	<.001	.41

^a^Pairs compare scores for each question before playing (b) and after playing (a) the game.

In the pre-evaluation, before students began using ISGs to develop their creativity and social competence, the mean score was 3.9. In the postevaluation, the mean score of the same group of students was 4.1. The student survey showed improvements in all aspects. The biggest improvement was observed in how well students believed they could do their homework using ISGs, and with regard to their general attitude toward schoolwork and solving problems by using such games. The difference between the prepilot and postpilot evaluation results was not as great as it was in the initial evaluation. However, the test group in the initial evaluation was more homogeneous, the students were not used to playing ISGs as part of the learning process, and, above all, the basic goal of the initial evaluation was to check motivation and concentration. In addition, the subsequent pilot evaluation already considered the findings of the initial evaluation. The applied improvements, as confirmed by the results, were successful.

By successfully using ISGs, students improved their social competence and creativity by enhancing the followings skills: (1) self-esteem and self-confidence, (2) motivation to participate and learn new things, (3) positive attitude toward teamwork with peers and teachers, (4) communication and collaboration with peers and teachers, (5) problem solving ability and enhanced creative thinking (solving problems creatively), (6) classroom performance, and (7) benefits for the classroom environment (teamwork).

## Discussion

### Principal Findings

Two aspects of ISGs cannot be overemphasized. First, children with disabilities (in the pilot study, children with intellectual disabilities) need appropriately developed ISGs. Second, these games and the pertinent didactic methodology should be based on an interoperable curriculum, so that teachers and trainers and children with disabilities can use them across multiple learning situations for developing creativity and cognitive and social competences. The scholarly literature on ISGs refers mostly to mainstream learning situations and often overlooks the possible benefits of implementing such games in the educational process of children and adolescents with learning disabilities and other impairments. On the other hand, numerous practices and activities are undetected and unexplained in analyses of game-based learning in education.

The results of the ISG4competence project show how to combine the knowledge, science, and practice of ISGs for learning with the science and practices from the field of pedagogy for children with disabilities. The project succeeded in developing social and cognitive competences in children with disabilities through a didactic approach using specially developed ISGs. For this purpose, we performed a careful needs analysis of the targeted population and educational context in all participating countries. We defined the curricular of ISGs according to the results of this survey. The 10 games developed as part of the project were successfully implemented.

The ISGs’ impact on social and cognitive competences was carefully observed during implementation and recorded with the help of a specially developed checklist for each competence defined in the game curricula. The final criterion for the decision regarding whether the games had an impact on social and cognitive competences of children with disabilities (people with intellectual disabilities or learning difficulties) was a comparison of the results of the pre-evaluation and postevaluation Likert-scale survey for social competences. This comparison showed remarkable progress in communication skills among the targeted population involved in the implementation of these games. Children included in the sample demonstrated progress in their communication competence and creativity, which was observed through their interaction with peers and teaching staff. To communicate with them, these children used both verbal and nonverbal channels. Their language messages were coherent with their body language; they used smiles, waves, and nods, and made eye contact with the addressees more often than before. With improved communication skills, self-esteem also increased in children who participated in the pilot phase of implementing these games. They became more assertive and aware of their own rights and needs, while expressing their wishes and preferences more clearly and appropriately. The competence of finding and using proper argumentation in a communication situation, in order to achieve the primary goal of the given communication act, was improved remarkably, which can be interpreted as a higher level of creativity in the students. Improvements in self-esteem and motivation were also observed in the students’ attitude toward their peers. After playing ISGs, children were more likely to expect a positive response while approaching others. In addition, their problem-solving competence was improved. Children were not as easily intimidated by bullying; they were able to express their frustrations and anger without escalating disagreements or harming others. Instead, they entered ongoing discussions by expressing relevant arguments and solutions, which is indicative of a growing competence for teamwork. In addition, the progress in their communication competence became a reflection of the children’s general social competence. More often than before, these children were found entering the social environment and gaining access to an ongoing group involved in either play or work. Moreover, they were successful in building a positive relationship with (1 or 2) peers, demonstrating progress in showing their capacity to care about them, and expressing that they missed their new friends if they were absent.

### Conclusion

In the ISG4competence project, the development of digital teaching and learning products focused on the didactic aspect. The proposed ISGs generated dynamic learning opportunities, engaging students in productive classroom discussion by encouraging them to become engaged, to argue, and to reflect on the learning goals. The games developed in this project are applicable as a means of support for education and training in varied educational settings: classrooms in mainstream schools, extracurricular activities, private lessons, private sessions with resource tutors, sessions with psychologists or speech therapists, or activities related to adolescent volunteering and informal groups.

ISGs for persons with disabilities, specifically for those who are integrated into mainstream education, should provide an opportunity for personalized learning, and should be tailored to ensure that every learner achieves the highest standard possible. However, we recommend that the games be adapted based on student needs and capabilities.

The ideas and results of the ISG4competence project could also serve as the basis for a longitudinal study of the qualitative assessment of the project with more end users.

## Appendix

Multimedia Appendix 1 Survey questionnaire.

## Abbreviations

ISG: intelligent serious game
ISG4competence: Intelligent Serious Games for Social and Cognitive Competence
TEIQue-SF: Trait Emotional Intelligence Questionnaire-Short Form
