# Supplementary material for: Development and Evaluation of Intelligent Serious Games for Children With Learning Difficulties: Observational Study
Source: JMIR Serious Games. 2020 Apr 16;8(2):e13190. doi: 10.2196/13190 (PMC7193434; doi:10.2196/13190)
Supplement: Multimedia Appendix 1 [file games_v8i2e13190_app1.docx]

Survey questionnaire

The first set of questions referred to the different ways in which students behave in school.

| Q1 | I can wait in line patiently. |
| --- | --- |
| Q2 | I sit still when I’m supposed to. |
| Q3 | I can wait for my turn to talk in class. |
| Q4 | I can easily calm down when excited. |
| Q5 | I calm down quickly when I get upset. |

The second set of questions referred to how well students feel they can do their schoolwork using intelligent serious games.

| Q6 | I can do even the hardest homework if I try. |
| --- | --- |
| Q7 | I can learn the things taught in school. |
| Q8 | I can figure out difficult homework. |

These next set referred to the way students get their work done.

| Q9 | If a solution to a problem is wrong the first time, I just keep trying until I get it right. |
| --- | --- |
| Q10 | I try to find a solution |
| Q11 | When I do badly on a test, I work harder the next time. |
| Q12 | I always work hard to complete my school work. |

The last set of questions referred to how students generally feel about school.

| Q13 | I do my schoolwork because I like to learn new things. |
| --- | --- |
| Q14 | I do my schoolwork because I’m interested in it. |
| Q15 | I do my schoolwork because I enjoy it. |
| Q16 | I like to solve problems by using games |
